# Creativity and psychopathology – an interdisciplinary view

**DOI:** 10.1192/j.eurpsy.2021.407

**Published:** 2021-08-13

**Authors:** R.M. Holm-Hadulla

**Affiliations:** Psycho-social Medicine, Heidelberg University, Heidelberg, Germany

**Keywords:** Creativity, psychopathology, Creative Bipolarity, psychotherapy

## Abstract

**Introduction:**

Since ancient philosophy extraordinary creativity is associated with mental disorders, emotional and cognitive destabilization, and melancholia. We here summarize the results of empirical and narrative studies and analyze most prominent cases of highly creative persons who suffered from depression, bipolar and schizotypic disorders, drug- and alcohol addiction. Hereby, we focus on the interaction of creative processes with “bipolar” personality traits. Finally, we offer an interdisciplinary interpretation of the creative dialectics between order and chaos.

**Objectives:**

An interdisciplinary concept of the relationship between creativity and psychopathology is shown to be essential for reasonable psychopharmacological and psychotherapeutic treatment of creative individuals.

**Methods:**

On the basis of empirical-statistical and biographical studies we offer a comprehensive concept of the interaction between creativity and psychopathology.

**Results:**

The exemplary cases of J. W. v. Goethe and Robert Schumann show a complex interaction of mood swings with creative achievements. Dysthymic and mild depressive phases were associated with creative efforts whereas severe depressive episodes inhibited their creativity. Mild mood swings and “bipolar personality traits” interacted constructively with their creative striving. With respect to the relationship of alcohol- and drug-abuse, we show on behalf of a detailed analysis of the life and work of prominent Pop-Icons that addiction mostly leads to psycho-social disintegration and destruction of creativity.

**Conclusions:**

An interplay between cognitive coherence and incoherence, emotional stability and instability, order and chaos accompanies many creative processes. The interdisciplinary approach shows that psychopathology can motivate creative efforts. However, if expressed severely, mental disorders inhibit or even destroy creativity.

**Disclosure:**

No significant relationships.

